# Targeting Bioenergetic, Redox and Prostaglandin Pathways in Long COVID-Associated Post-Exertional Malaise and Brain Fog: A Nutraceutical Translational Hypothesis

**DOI:** 10.3390/nu18162650

**Published:** 2026-08-13

**Authors:** Stephan F. E. Praet

**Affiliations:** 1 University of Canberra Research Institute for Sport and Exercise (UCRISE), University of Canberra, Canberra ACT 2617, Australia; research@drstephanpraet.com.au; 2 Ochre Health Medical Centre Bruce, University of Canberra, Canberra ACT 2617, Australia

**Keywords:** Long COVID, post-acute sequelae of SARS-CoV-2, post-exertional malaise, brain fog, mitochondrial dysfunction, oxidative stress, Nrf2, mPGES-1, nutraceuticals, coenzyme Q10

## Abstract

Post-exertional malaise (PEM) and cognitive dysfunction (hereafter “cognitive dysfunction”, including the patient-reported syndrome often described as “brain fog”) are among the most disabling features of Long COVID; yet, approved disease-modifying treatments remain lacking. Emerging evidence implicates interacting disturbances in mitochondrial bioenergetics, redox regulation and neurovascular inflammation, although much of the supporting evidence remains indirect and derives from acute COVID-19, myalgic encephalomyelitis/chronic fatigue syndrome (ME/CFS), primary mitochondrial disease, inflammatory biology and mechanistic pharmacology rather than from direct Long COVID intervention trials. This hypothesis-generating narrative review develops a mechanism-based translational framework: that a pathway-targeted nutraceutical programme may modulate selected elements of these three axes, subject to prior demonstration of formulation quality, pharmacokinetic feasibility, target engagement and safety. Candidate modules comprise coenzyme Q10 and alpha-lipoic acid for bioenergetic/redox support; selenium, sulforaphane and resveratrol for Nrf2–thioredoxin-related redox regulation; and *Boswellia serrata*, luteolin and eicosapentaenoic acid for putative prostaglandin/resolution-pathway modulation. Sonlicromanol provides a conceptual mechanistic precedent for combined redox and prostaglandin-directed pharmacology, but it is not considered pharmacologically equivalent to an eight-agent nutraceutical combination. We summarise the mechanistic rationale, distinguish direct from indirect evidence, define qualitative evidence-grading criteria, outline safety and interaction considerations, and propose a staged translational research programme. This framework is intended to generate falsifiable hypotheses for future Long COVID studies, not to imply established clinical efficacy.

## 1. Introduction

Long COVID—formally the post-acute sequelae of SARS-CoV-2 infection (PASC) or post-COVID-19 condition—has been estimated to affect approximately 6–10% of infected individuals in population-level analyses, although estimates vary substantially according to case definition, vaccination/variant era, ascertainment method and duration of follow-up [1,2]. Among its heterogeneous manifestations, post-exertional malaise (PEM) and cognitive dysfunction (including subjective “brain fog” and objectively measurable deficits in attention, processing speed or executive function) are consistently ranked by patients as among the most disabling symptoms and are strongly associated with failure to return to work and reduced quality of life [3]. PEM—a disproportionate and delayed worsening of symptoms after physical, cognitive or emotional exertion—is the cardinal feature shared with myalgic encephalomyelitis/chronic fatigue syndrome (ME/CFS), and its presence constrains fixed-increment graded-exercise rehabilitation approaches [4].

Despite intense investigation, no licenced disease-modifying therapy exists for these symptoms. Symptomatic and pacing-based strategies remain the mainstay; yet, they neither reverse the underlying pathophysiology nor restore exercise tolerance [5]. This therapeutic vacuum, combined with an increasingly coherent but incomplete mechanistic picture, creates a rational opportunity to test interventions that may engage putative downstream pathways associated with PEM and cognitive dysfunction.

Three pathological processes have emerged repeatedly and, importantly, may be mechanistically interconnected rather than independent: (i) mitochondrial dysfunction and bioenergetic insufficiency; (ii) a self-sustaining redox imbalance with impaired endogenous antioxidant defence; and (iii) persistent low-grade neurovascular inflammation [6,7]. Each may be amenable, at least in principle, to modulation by specific nutraceutical agents with plausible molecular targets. The purpose of this hypothesis-generating narrative review is to move beyond the generic framing of nutraceuticals as undifferentiated “antioxidants” and instead articulate a pathway-specific framework: that a rationally selected nutraceutical strategy may modulate these axes and generate testable hypotheses for attenuating PEM and cognitive dysfunction. We develop the pathophysiological framework, describe the search and selection process, map candidate agents to molecular targets, appraise direct and indirect clinical evidence using explicit qualitative criteria, address safety, and propose a staged translational research programme rather than a definitive efficacy trial.

## 2. Search Strategy, Evidence Selection and Candidate Selection

This narrative review was developed from targeted searches of PubMed/MEDLINE, Scopus and Google Scholar, supplemented by reference-list screening and forward citation tracking of key papers. The final search was completed on 6 July 2026. Searches were restricted to English-language publications. Human Long COVID studies, randomised controlled trials, systematic reviews, clinical pharmacokinetic studies and mechanistic studies were prioritised; animal, cell-culture and non-Long COVID disease literature was included only when direct Long COVID evidence was absent and when the evidence addressed a specific mechanistic link or formulation issue. The review was planned and revised using the transparency domains of SANRA (justification of importance, statement of aims, literature-search description, referencing, scientific reasoning and appropriate presentation of data), while recognising that this was not a systematic review and did not use PRISMA flow counting, formal risk-of-bias scoring or quantitative pooling.

Principal Boolean strings included: (“Long COVID” OR “post-COVID condition” OR PASC OR “post-acute sequelae of SARS-CoV-2”) AND (“post-exertional malaise” OR PEM OR fatigue OR “exercise intolerance” OR “brain fog” OR “cognitive dysfunction”); (“Long COVID” OR PASC) AND (mitochondria* OR “oxidative phosphorylation” OR “electron transport chain” OR “pyruvate dehydrogenase” OR PDH OR lactate OR “oxygen extraction”); (“Long COVID” OR PASC OR COVID-19) AND (“oxidative stress” OR ROS OR Nrf2 OR NRF2 OR Keap1 OR thioredoxin OR “thioredoxin reductase” OR peroxiredoxin OR glutathione); (“Long COVID” OR PASC) AND (endothelial OR neurovascular OR “blood–brain barrier” OR microclot* OR NF-κB OR COX-2 OR mPGES-1 OR “microsomal prostaglandin E synthase” OR PGE2 OR prostaglandin); and (“Long COVID” OR PASC OR fatigue OR “cognitive dysfunction”) AND (“coenzyme Q10” OR ubiquinol OR ubiquinone OR “alpha-lipoic acid” OR selenium OR sulforaphane OR glucoraphanin OR resveratrol OR Boswellia OR AKBA OR luteolin OR EPA OR “eicosapentaenoic acid” OR omega-3).

Candidate compounds were selected through an iterative mechanistic screen rather than a formal systematic-review ranking; the criteria used to retain or deprioritise agents are stated explicitly in Section 4. Agents were retained when they had: (1) a plausible mechanistic link to one of the three axes; (2) at least some human clinical, safety or pharmacokinetic literature; (3) feasible oral delivery or a clearly definable formulation requirement; (4) measurable exposure or target-engagement biomarkers; (5) chronic-administration safety issues that could be protocolised; and (6) a manageable rationale within a staged combination programme. Agents were deprioritised if they lacked human-use data, required parenteral delivery, had poorly standardised preparations, had unacceptable interaction risk for a Long COVID population, or duplicated a mechanism already represented by a better-characterised candidate.

Because search yields overlapped heavily across databases and the review was not registered as a systematic review, records were not counted in PRISMA fashion. For transparency, the working bibliography used for revision contained approximately 300 title/abstract-level records, approximately 90 full-text or full-record evaluations, and 64 references selected for citation after removal of duplicates, non-English records, inaccessible abstracts, low-relevance mechanistic studies and papers not materially informing the proposed framework. These counts should be interpreted as approximate narrative-review audit figures rather than systematic-review screening numbers.

## 3. A Three-Axis Pathophysiological Framework

### 3.1. Mitochondrial Dysfunction and Bioenergetic Failure

SARS-CoV-2 interacts intimately with host mitochondria. Viral open reading frame and non-structural proteins localise to mitochondrial membranes, disrupting electron transport chain (ETC) assembly, promoting pathological fission, and provoking calcium dyshomeostasis with opening of the mitochondrial permeability transition pore [7,8]. A plausible downstream consequence, supported by the related post-viral and ME/CFS literature, is functional restriction of pyruvate dehydrogenase (PDH) flux at the irreversible gateway converting pyruvate to acetyl-CoA. Oxidative modification of the PDH lipoyl cofactor, together with upregulation of PDH kinase, is proposed to force cells toward aerobic glycolysis—a Warburg-like shift that yields only two ATP per glucose molecule versus approximately 30–32 through oxidative phosphorylation, with attendant lactate accumulation [8,9,10].

Plasma metabolomic signatures in Long COVID support impaired fatty-acid oxidation, perturbed tricarboxylic-acid-cycle intermediates and reduced Complex I activity [8]. Additional PBMC and tissue/biomarker studies published in 2025 further support persistent mitochondrial abnormalities in Long COVID, including altered mitochondrial ATP-synthase behaviour, ultrastructural changes and disrupted mitophagy markers [11,12]. This bioenergetic insufficiency is present at rest but is unmasked catastrophically by exertion: invasive cardiopulmonary exercise testing demonstrates impaired systemic oxygen extraction rather than a primarily cardiopulmonary limitation [5]. Muscle biopsy studies further show that exercise precipitates mitochondrial and structural myofibre abnormalities specifically in the post-exertional window, providing a direct histological correlate of PEM [4]. The mechanistic congruence with ME/CFS—shared PDH blockade, glycolytic shift and exertional deterioration—strengthens the case for targeting mitochondrial bioenergetics [10].

### 3.2. Redox Imbalance and the Nrf2–Thioredoxin Axis

Reactive oxygen species (ROS) may be generated in excess through ETC electron leak, NADPH-oxidase activation and uncoupled nitric oxide synthase, producing superoxide, hydrogen peroxide and peroxynitrite [13]. Long COVID cohorts have shown oxidative-damage and redox-imbalance markers, including malondialdehyde/protein-carbonyl-related oxidative toxicity and elevated oxidative-stress index in patients with fatigue and cognitive symptoms [14,15]. Mechanistically, acute or moderate oxidative/electrophilic stress can activate Nrf2 through Keap1 cysteine modification; in contrast, direct SARS-CoV-2 interference with NRF2 signalling has been demonstrated in experimental infection systems [16]. Whether this viral interference persists as genuine Nrf2 suppression in established Long COVID remains unproven. A more cautious interpretation is that chronic Long COVID may involve inadequate, exhausted or dysregulated antioxidant-response capacity relative to ongoing oxidative burden. Under physiological conditions, Nrf2 released from Keap1 drives expression of cytoprotective genes including thioredoxin reductase-1 (TrxR1), NAD(P)H quinone oxidoreductase 1 (NQO1) and glutamate-cysteine ligase (GCL) [17,18].

The thioredoxin reductase–thioredoxin–peroxiredoxin (TrxR–Trx–Prdx) system is one major enzymatic network for maintaining thiol redox status and supporting peroxide detoxification, but it should not be considered the only peroxide-clearing pathway; glutathione peroxidases and catalase also contribute substantially [19,20]. TrxR maintains thioredoxin in its reduced state, thereby supporting peroxiredoxin activity and other thioredoxin-dependent redox reactions. Because TrxR is a selenoenzyme and oxidative stress may impair redox enzymes, selenium status and Nrf2-regulated antioxidant capacity provide a plausible, but not yet proven, interface between redox biology and Long COVID chronicity. The proposed redox–energetic loop should therefore be read as a testable model rather than an established causal sequence [19,21].

### 3.3. Neurovascular Inflammation and a Hypothesised mPGES-1/PGE_2_ Contribution

Endothelial injury—via spike–ACE2 interactions, complement activation and neutrophil extracellular-trap formation—may converge on nuclear factor kappa-B (NF-κB) activation in endothelium and macrophages [22,23]. Persistent endothelial dysfunction has been directly documented in Long COVID cohorts using non-invasive vascular assessment, confirming that vascular injury can outlast the acute infectious phase [24]. NF-κB activation can induce pro-inflammatory cytokines and prostaglandin-biosynthetic enzymes such as cyclooxygenase-2 (COX-2) and microsomal prostaglandin E_2_ synthase-1 (mPGES-1). Persistence of spike protein within CD16^+^ monocytes for up to fifteen months provides a plausible mechanism for sustained immune activation in a subset of patients, although the degree to which this specifically produces an mPGES-1/PGE_2_ phenotype in Long COVID remains unknown [25].

mPGES-1 is the terminal, inducible enzyme converting the COX-2 product PGH_2_ to prostaglandin E_2_ (PGE_2_) [26]. Unlike non-selective COX inhibition, selective mPGES-1 modulation is theoretically attractive because it may reduce inducible PGE_2_ generation while sparing other prostanoids. However, the Long COVID evidence for this pathway is indirect. PGE_2_ biology is context-, tissue- and EP-receptor-dependent: EP1/EP3 signalling can contribute to nociception, fever and vasomotor effects, EP2/EP4 signalling may be vasodilatory and immunomodulatory, and both pro-inflammatory and resolution-associated effects may occur depending on timing, compartment and receptor expression [26,27]. Peripheral endothelial dysfunction, systemic inflammation and blood–brain-barrier disruption in Long COVID support a neurovascular inflammatory framework [24,28], but they do not demonstrate a PGE_2_-specific abnormality. Alternative inflammatory and lipid-mediator pathways—including leukotrienes, thromboxanes, prostacyclin, complement, cytokines, mast-cell mediators and specialised pro-resolving mediators—should therefore remain in the differential mechanistic model [27,29]. Central or systemic prostanoid measurements, including plasma/urinary PGE_2_ metabolites and, where ethically justified, cerebrospinal-fluid prostanoid profiling or neuroimaging markers, should be treated as exploratory hypothesis-testing biomarkers rather than assumed proof of pathway activation.

Recent CSF proteomic data in ME/CFS provide additional indirect support for a central neuroimmune and thrombo-inflammatory component relevant to this framework. Bragée et al. quantified 902 proteins in CSF from 31 ME/CFS patients and reported POTS-associated enrichment of platelet-related, neutrophil-degranulation and acute-phase-response pathways, while severe disease was associated with complement cascade, fibrin-clot formation and IGFBP-mediated insulin-like growth factor transport. Ratio-based analyses further identified severity-associated protein pairs spanning cellular stress, extracellular remodelling and immune-neuronal interaction. These findings do not establish a Long COVID-specific or mPGES-1/PGE_2_-specific mechanism, nor do they test nutraceutical interventions, but they strengthen the indirect ME/CFS evidence that PEM/cognitive-dysautonomic phenotypes can be accompanied by central immune, vascular/coagulation, metabolic-neurotrophic and neuronal-stress signatures [30].

### 3.4. Convergence on Post-Exertional Malaise and Brain Fog

**Figure 1:**
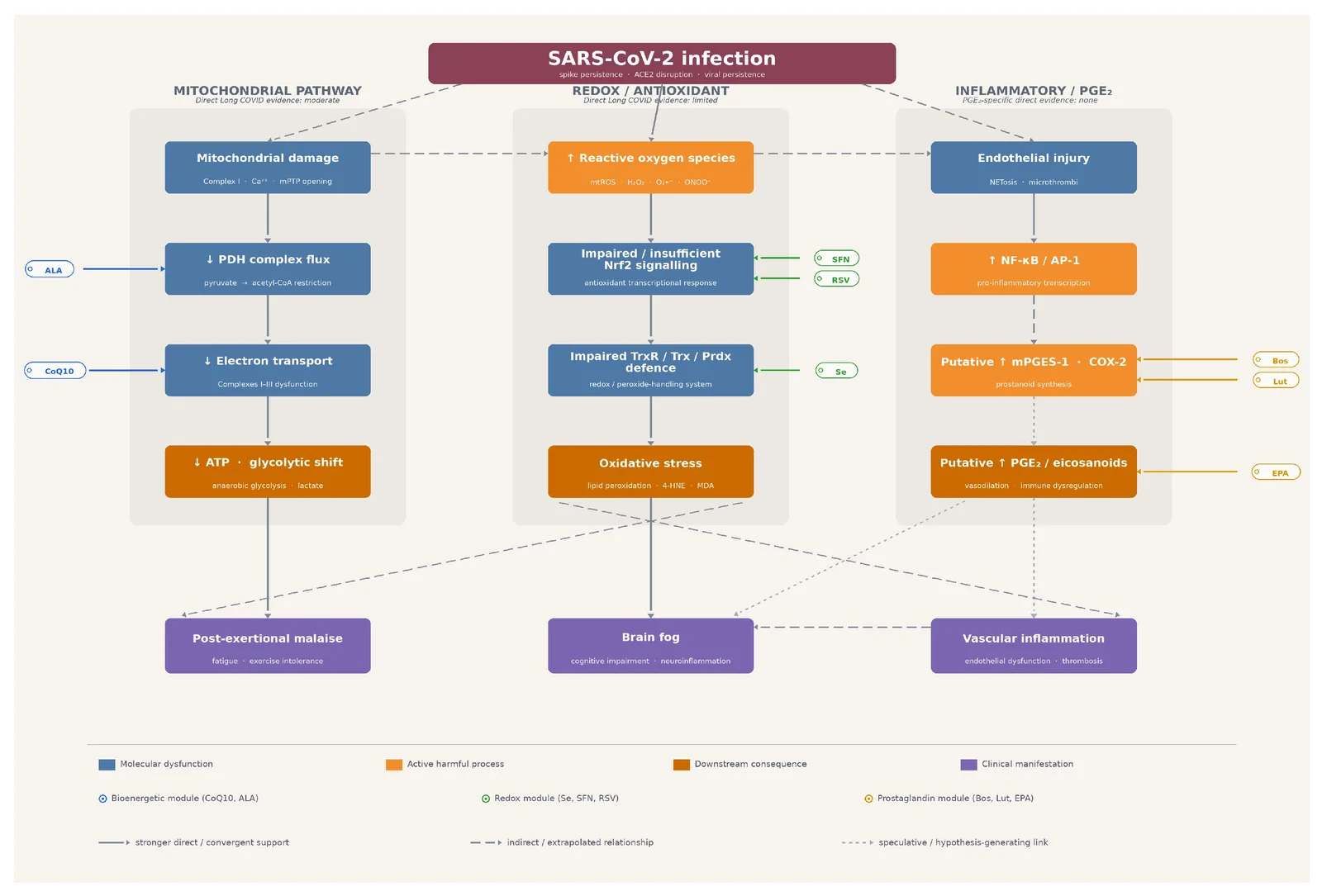
Three-axis pathophysiological model of post-exertional malaise and brain fog in Long COVID, showing proposed nutraceutical intervention sites. The three pathway panels are annotated with the relative level of direct Long COVID evidence (moderate for the mitochondrial pathway, limited for the redox/antioxidant pathway, with none for the specific claim of persistent Nrf2–thioredoxin suppression, and none for the PGE_2_-specific inflammatory arm). SARS-CoV-2 is proposed to drive three interlocking cascades—mitochondrial (left), redox/antioxidant (centre) and inflammatory/prostaglandin (right)—that converge on the clinical manifestations (bottom row). Solid grey arrows denote relationships with stronger direct or convergent support; dashed grey arrows denote indirect or extrapolated relationships; and dotted grey arrows denote more speculative, hypothesis-generating links. Coloured tags mark the proposed intervention site of each candidate agent, grouped as the bioenergetic module (blue: CoQ10, alpha-lipoic acid [ALA]), the redox module (green: selenium [Se], sulforaphane [SFN], resveratrol [RSV]) and the prostaglandin module (amber: Boswellia [Bos], luteolin [Lut], eicosapentaenoic acid [EPA]); arrowheads indicate metabolic support/activation and bars indicate enzymatic inhibition. The figure is conceptual and does not imply that each agent acts exclusively, or with proven clinical efficacy, at the indicated node in Long COVID; see Table 1 for the fuller target profile of each agent. COX-2, cyclooxygenase-2; mPGES-1, microsomal prostaglandin E synthase-1; mPTP, mitochondrial permeability transition pore; Nrf2, nuclear factor erythroid 2-related factor 2; PDH, pyruvate dehydrogenase; PGE_2_, prostaglandin E_2_; TrxR, thioredoxin reductase.

These axes are proposed to be interlocking rather than parallel (Figure 1). Mitochondrial dysfunction may increase ROS production; an inadequate or dysregulated antioxidant response may permit oxidative stress to persist; oxidative and inflammatory signalling may amplify NF-κB-related cytokine and lipid-mediator pathways; and neurovascular inflammation may further impair microvascular oxygen delivery and mitochondrial function. PEM may emerge when exertion acutely increases metabolic demand upon a system with limited bioenergetic reserve, precipitating an oxidative–inflammatory flare. Cognitive dysfunction may reflect related processes expressed within a neuroinflamed or barrier-compromised central compartment. A strategy that engages multiple axes is therefore hypothesised to be more informative for translational testing than antioxidant monotherapy, but this remains unproven [6,7,31].

**Table 1:** Candidate nutraceuticals proposed molecular targets, selection rationale, formulation/exposure constraints, indicative research-dose ranges and principal safety limitations.

Agent	Target/Rationale	Clinical Evidence Level	Formulation/ Exposure Criteria	Indicative Research Dose/Source	Principal Safety Limitations
Coenzyme Q10	ETC redox carrier; bioenergetic support, not proven to reverse Long COVID mitochondrial pathology.	Direct Long COVID monotherapy RCT negative; combination evidence indirect through CoQ10 + ALA study.	Formulation-dependent; ubiquinol or solubilised ubiquinone acceptable if plasma CoQ10 exposure is documented.	100–200 mg twice daily. Lower bound as administered in the CoQ10 + ALA chronic-COVID study (100 mg twice daily, ubiquinone) [32]; upper bound a proposed research extension informed by CoQ10 supplementation and exposure literature [33].	Warfarin interaction potential; GI effects; baseline status/exposure variability.
Alpha-lipoic acid	Redox-active/metabolic modulator; no claim that oral ALA restores covalently attached PDH lipoylation.	Direct evidence only as part of CoQ10 + ALA non-randomised comparison; metabolic evidence indirect.	Specify R/S form if relevant; monitor glucose and tolerability.	100–200 mg twice daily. Lower bound as administered in the CoQ10 + ALA chronic-COVID study (100 mg twice daily) [32]; upper bound a proposed research extension informed by ALA pharmacology and human supplementation literature [34,35].	May lower glucose; caution with diabetes, glucose-lowering therapy and hypoglycaemia risk.
Selenium	Selenoenzyme support for TrxR and glutathione peroxidases; status-guided redox support.	No direct Long COVID efficacy evidence; strong nutritional biology but indirect.	Measure baseline selenium; avoid supplementation in selenium-replete/high-intake participants.	100–200 µg/day, status-guided supplementation informed by the selenium nutritional literature [36] and constrained by the EFSA adult tolerable upper intake level [37]. Supplement only where baseline status and the applicable jurisdictional upper limit permit.	Narrow therapeutic window; adult UL differs by jurisdiction; chronic excess risk.
Sulforaphane/glucoraphanin	Nrf2/Keap1 pathway activator; exposure depends on myrosinase conversion.	No direct Long COVID efficacy evidence; human exposure data and mechanistic support indirect.	Distinguish free sulforaphane from glucoraphanin; report molar equivalents; verify urinary/plasma metabolites.	10–40 mg/day free SFN equivalent, or a defined molar glucoraphanin + myrosinase equivalent. Proposed phase 0/1 range informed by human sulforaphane exposure and glucosinolate-conversion literature [38,39]; not derived from formal dose-ranging studies.	GI effects; interaction claims remain uncertain; medication review required.
Resveratrol	Nrf2/NF-κB/SIRT1-related signalling; possible redox-inflammatory bridge.	No direct Long COVID efficacy evidence; human intervention and PK literature indirect.	Formulation-specific PK needed; micronised/phytosome/lipid systems not interchangeable.	150–500 mg/day, only with formulation justification and exposure data. Range summarised from published human intervention trials of resveratrol [40] rather than from a dedicated dose-ranging study.	Antiplatelet potential; CYP/UGT interaction uncertainty; GI effects.
*Boswellia serrata*	Gum-resin boswellic acids including AKBA; in vitro mPGES-1/NF-κB-related rationale.	No Long COVID evidence; osteoarthritis clinical data do not prove mPGES-1 mediation.	Specify botanical name, plant part, extract ratio, AKBA/total boswellic acids, contaminants and stability.	100–300 mg/day standardised extract, only if standardisation and PK rationale are provided. Doses of 100 and 250 mg/day of a 30% AKBA-standardised extract were administered in a randomised osteoarthritis trial [41]; the 300 mg/day upper bound is a proposed extension beyond the highest dose tested.	Antiplatelet/GI potential; product variability; allergy/contaminant risk.
Luteolin	Flavonoid with COX-2/mPGES-1 and broader inflammatory signalling effects in models.	No direct Long COVID efficacy evidence; human translation limited by low bioavailability.	Formulation-specific PK required; glucuronidation/sulfation prominent; delivery systems not interchangeable.	Human research dose not established. Formulation-specific phase 0/1 dose escalation with pharmacokinetic confirmation is required before an adult research range can be proposed; preclinical anti-inflammatory and mechanistic work [42,43] does not constitute human dose-ranging evidence.	GI effects; interaction uncertainty; exposure may be insufficient with raw powder.
EPA	Eicosanoid substrate shift and precursor for specialised pro-resolving mediators.	Long COVID omega-3 evidence feasibility only; high-dose EPA evidence indirect from cardiovascular/inflammatory literature.	Prefer EPA-dominant, pharmaceutical-quality product; specify DHA content/absence, oxidation and purity.	2–4 g/day EPA proposed as a target-engagement range, not a proven Long COVID dose. The 4 g/day upper bound is derived from pharmaceutical icosapent ethyl data [44] and should not be extrapolated directly to non-pharmaceutical EPA formulations; anti-inflammatory dose rationale is informed by the eicosanoid literature [45].	Bleeding and atrial fibrillation/flutter considerations; oxidation/purity critical.

The “Indicative research dose/source” column distinguishes three levels of provenance: doses actually administered in identifiable human studies; ranges summarised from human intervention or exposure literature; and ranges proposed for phase 0/1 evaluation only. Where no adequate human dose-ranging evidence exists, this is stated rather than a numerical range being implied. Doses are proposed for staged hypothesis-driven clinical research and should not be interpreted as individual medical advice. Final dosing requires protocol-specific pharmacokinetic review, medication-interaction screening, batch-specific product-quality verification, exposure biomarker confirmation where feasible and independent safety oversight. AKBA, acetyl-11-keto-β-boswellic acid; COX-2, cyclooxygenase-2; EPA, eicosapentaenoic acid; ETC, electron transport chain; mPGES-1, microsomal prostaglandin E_2_ synthase-1; Nrf2, nuclear factor erythroid 2-related factor 2; PDH, pyruvate dehydrogenase; TrxR, thioredoxin reductase.

### 3.5. Relationship to Alternative Pathophysiological Models

The three-axis framework advanced here is not offered as a competitor to, still less a replacement for, the other major pathophysiological models of Long COVID, and it would misrepresent the present evidence to claim that it is better supported than they are. Four alternative models command substantial and, in several respects, stronger direct human evidence. The viral persistence model holds that SARS-CoV-2 RNA, protein or replication-competent virus persists in tissue reservoirs beyond the acute illness and continuously stimulates the immune system; this model has direct tissue-level human support and has advanced far enough to generate antiviral and monoclonal-antibody trials in Long COVID [46]. The autoimmunity model posits that infection triggers functional autoantibodies, notably agonistic autoantibodies against G-protein-coupled receptors including β2-adrenergic, muscarinic M2, angiotensin II AT1 and MAS receptors, which have been detected in patients with persistent post-COVID symptoms [47]. The autonomic dysfunction model attributes exercise intolerance, orthostatic symptoms and cognitive complaints to dysautonomia, including post-COVID POTS and related autonomic syndromes [48]; this partly converges with the autoimmunity model, since several implicated GPCR targets are themselves autonomic receptors [47]. The broader immune-dysregulation model encompasses herpesvirus reactivation, T-cell exhaustion, altered cortisol dynamics and microbiome disturbance, and is supported by cross-sectional immune-profiling studies [49].

Rather than competing with these models, the framework proposed here is best understood as describing a possible downstream convergence point. Viral antigen persistence, autoantibody engagement of GPCRs and chronic immune activation are all plausible upstream initiators of precisely the mitochondrial, redox and prostaglandin disturbances described in Section 3.1, Section 3.2 and Section 3.3; conversely, none of the three axes proposed here offers any account of why the process begins, or why it persists in some individuals and not others, which is what the upstream models supply. This complementarity has consequences that matter for trial design. Because a downstream metabolic intervention would not be expected to eliminate a persistent viral reservoir or to remove pathogenic autoantibodies, the framework predicts at most symptomatic and functional benefit rather than disease resolution, and predicts that benefit would be greatest in patients in whom the upstream driver has subsided but the downstream metabolic disturbance has not. It follows that any trial of the proposed combination should measure, or at minimum stratify for, markers of these alternative mechanisms, since a null result in an unstratified cohort could reflect aetiological heterogeneity rather than failure of the intervention. The framework is also falsifiable against these alternatives: if post-exertional malaise and cognitive dysfunction were shown to track antigen persistence or autoantibody titre independently of bioenergetic and redox markers, the case for a clinically meaningful downstream metabolic contribution would be correspondingly weakened.

## 4. Nutraceutical Candidates and Their Molecular Targets

Table 1 summarises the eight candidate agents, their principal molecular targets, selection rationale, formulation/exposure constraints, proposed research-dose ranges and safety limitations. The compounds were retained only when they met several explicit translational criteria, applied iteratively during candidate selection: mechanistic relevance to one of the three axes; at least some human use or clinical data; plausible oral pharmacokinetic feasibility or a defined formulation requirement; product standardisation potential; chronic-administration safety constraints that can be protocolised; and compatibility with a staged trial programme. Several agents act across more than one axis, but none should be interpreted as pathway-specific in the pharmacological sense.

### 4.1. Mitochondrial Support: Coenzyme Q10 and Alpha-Lipoic Acid

Coenzyme Q10 (CoQ10) is an obligatory lipophilic electron carrier shuttling electrons between Complexes I/II and III; supplementation can increase circulating CoQ10 and may support ETC redox cycling, although clinical response depends strongly on formulation, absorption and baseline status [33]. Alpha-lipoic acid (ALA) is best framed here as a redox-active and metabolic modulator rather than as a direct replacement for the covalently attached PDH lipoyl cofactor. The functional lipoyl group of mammalian PDH is generated through mitochondrial lipoylation pathways and should not be assumed to be restored by orally administered free ALA. ALA may still be relevant through antioxidant actions, thiol redox effects and indirect metabolic signalling [34,35]. A prospective, non-randomised comparison of 174 patients reported greater fatigue improvement with CoQ10 plus ALA than in an untreated control group; whereas, a placebo-controlled trial of high-dose CoQ10 monotherapy over six weeks was negative [32,50]. These findings support clinical equipoise, not efficacy.

### 4.2. Redox Restoration: Selenium, Sulforaphane and Resveratrol

Selenium is incorporated as selenocysteine into the catalytic site of TrxR and glutathione peroxidases; adequate selenium status is a prerequisite for several antioxidant enzymes, but supplementation should be status-guided rather than indiscriminate [36]. Sulforaphane, the isothiocyanate generated by myrosinase-catalysed hydrolysis of glucoraphanin, is a well-characterised dietary Nrf2 activator, but potency and exposure depend on whether the intervention delivers free sulforaphane or glucoraphanin plus active myrosinase [38,39,51]. Any clinical protocol should express dose in free-sulforaphane equivalents or molar glucoraphanin/sulforaphane equivalents and verify exposure using urinary or plasma sulforaphane metabolites. Resveratrol can modulate Nrf2 and NF-κB-related signalling through SIRT1-associated and other mechanisms, but rapid phase-II metabolism and low systemic bioavailability make formulation-specific pharmacokinetic justification essential [40].

### 4.3. Prostaglandin Modulation: Boswellia, Luteolin and Eicosapentaenoic Acid

Boswellic acids—principally acetyl-11-keto-β-boswellic acid (AKBA) from the gum resin of *Boswellia serrata* Roxb. ex Colebr.—are among the natural compounds shown in vitro to inhibit mPGES-1, in addition to suppressing NF-κB/AP-1-related transcription [52]. This does not establish that clinical benefit in osteoarthritis or any other condition is mediated by mPGES-1 inhibition in humans [41]. Any trial should specify the botanical name, plant part, extraction procedure, extract ratio, analytical standardisation for AKBA/total boswellic acids, contaminant testing and stability. Luteolin reduces COX-2 and mPGES-1 expression in activated macrophage models and has broader anti-inflammatory effects, but human translation is constrained by rapid glucuronidation/sulfation and limited formulation-specific pharmacokinetic data [42,43]. EPA competitively displaces arachidonic acid in eicosanoid pathways and is a precursor to specialised pro-resolving mediators [45,53]. However, pharmaceutical icosapent ethyl data cannot be assumed to apply directly to generic EPA supplements or to Long COVID; an EPA-dominant intervention should specify EPA content, DHA content or absence, peroxide/anisidine oxidation values, purity, stability and batch certification. Anti-inflammatory and pro-resolving dose ranges are extrapolated from non-Long-COVID literature and require confirmation in target-engagement studies [44].

## 5. Integrated Hypothesis and Combination Rationale

We propose that because the three axes may be mutually reinforcing, a multi-module intervention could be more mechanistically informative than single-agent antioxidant monotherapy. This should not be read as proof that all three modules are necessary, sufficient or synergistic. A negative CoQ10 monotherapy trial may reflect the compound, formulation, dose, duration, population, endpoint or underlying biology rather than failure to engage enough axes [50]. The proposed combination is therefore conceived as a staged research framework comprising a bioenergetic/redox module (CoQ10 + ALA), a redox-response module (selenium + sulforaphane, with resveratrol as a bridging agent) and a putative prostaglandin/resolution module (Boswellia + luteolin + EPA), each of which requires independent feasibility and target-engagement testing.

Sonlicromanol (KH176) provides an informative conceptual mechanistic analogy, not pharmacological proof-of-concept for the proposed nutraceutical combination. Sonlicromanol is a defined small molecule with specific stereochemistry, pharmacokinetics, tissue distribution and metabolite-dependent target activity; the proposed nutraceutical agents differ markedly in exposure, selectivity, intracellular localisation and regulatory quality control [54,55]. In primary mitochondrial disease, sonlicromanol has progressed through phase 2 evaluation, but the phase 2b programme should be interpreted cautiously: the randomised dose-selection study did not meet its primary cognitive endpoint, although exploratory and open-label extension signals were reported [56,57]. Thus, sonlicromanol supports the plausibility of jointly considering redox and prostaglandin biology, but it does not imply that a food-derived multi-agent regimen can reproduce the drug’s activity.

## 6. Appraisal of Direct and Indirect Clinical Evidence

Direct clinical evidence in Long COVID remains sparse, and it is important to distinguish direct evidence for individual agents from evidence for the complete combination, which is currently absent. The strongest direct signal is for CoQ10–ALA: a prospective, non-randomised comparison of 174 patients reported fatigue improvement among recipients relative to an untreated control group [32]. This design is susceptible to expectation effects, confounding, differential care-seeking and natural-recovery bias and cannot establish efficacy. Instructively, a rigorously conducted placebo-controlled trial of CoQ10 monotherapy was negative over a six-week exposure in a predominantly overweight cohort [50]. The contrast is compatible with, but does not prove, the hypothesis that combination or multi-axis approaches may be necessary; the results may simply reflect differences in compound selection, dose, duration, baseline phenotype or outcome measurement.

The available Long COVID omega-3 study is best read as a feasibility trial: it primarily supports tolerability and recruitment feasibility while providing insufficient evidence for efficacy, particularly because it did not test a pharmaceutical-grade EPA-dominant anti-inflammatory strategy [58]. Indirect support for the broader framework is stronger but still non-definitive: antioxidant and anti-inflammatory nutritional strategies have a coherent rationale in COVID-19 pathophysiology [31,59]; ALA improves metabolic and oxidative parameters across multiple conditions without proving correction of PDH flux in Long COVID [60]; high-dose EPA has cardiovascular anti-inflammatory and safety data in selected high-risk populations [44]; and mitochondrial-disease literature provides a rationale for combined nutraceutical approaches while not validating this specific Long COVID combination [61]. The strength of evidence underpinning each principal claim is summarised in Table 2. Across the framework, direct human Long COVID evidence ranges from none to moderate, mechanistic and preclinical support varies by pathway, and overall certainty for the complete intervention is very low. Beyond the agents proposed here, the wider nutraceutical trial literature in Long COVID is small but not empty, and it is instructive. In a single-blind randomised placebo-controlled trial in 50 adults with Long COVID and persistent fatigue, 28 days of l-arginine (1.66 g twice daily) plus liposomal vitamin C (500 mg twice daily) improved the primary endpoint of 6 min walk distance, together with handgrip strength, flow-mediated dilation and fatigue persistence, relative to placebo [62]. That trial is relevant to the present framework in three respects: it demonstrates that an adequately powered nutraceutical trial in this population is feasible; its mechanism of action is vascular and nitric-oxide-mediated rather than bioenergetic, redox or prostaglandin-directed, so it tests a different hypothesis from the one advanced here; and its short duration, single-blind design, modest sample size and absence of post-exertional-malaise or objective cognitive endpoints limit what can be concluded from it. It establishes feasibility and provides encouragement for the field, not support for the specific combination proposed in this hypothesis-generating narrative review.

## 7. Safety, Interactions and Translational Considerations

A combination of eight agents raises real-world issues of adherence, tolerability, attribution of adverse effects, supplement contamination, batch variability and cumulative interactions, and these must be addressed explicitly before any trial. EPA at 2–4 g/day should be treated as pharmacologically distinct from low-dose “fish oil”; REDUCE-IT used pharmaceutical icosapent ethyl in a high-risk cardiovascular population and reported higher atrial fibrillation/flutter hospitalisation and numerically higher serious bleeding [44]. EPA, resveratrol and Boswellia may each carry antiplatelet potential, so concurrent anticoagulant use, high-risk NSAID use and bleeding disorders should be exclusion criteria in early trials. Selenium requires baseline-status logic: although many sources cite an adult upper limit near 400 µg/day, European authorities have adopted a lower adult tolerable upper intake level of 255 µg/day, meaning a 100–200 µg/day supplement can approach the European UL once diet is included [36,37]. ALA can lower blood glucose and requires glucose-risk screening in people with diabetes, on glucose-lowering therapy, or prone to hypoglycaemia [34,60]. CoQ10 may attenuate warfarin anticoagulation [33]. The previously suggested clinically relevant sulforaphane–CYP1A2 interaction has been removed because available human evidence is insufficient to support it as a material protocol restriction; nonetheless, narrow-therapeutic-index medication use should be reviewed case by case. Pregnancy, lactation, current anticoagulation and unstable comorbid disease should be explicit exclusions for a first-in-patient trial, and all products should undergo independent identity, purity, potency, oxidation, contaminant and stability testing.

## 8. Future Directions: A Proposed Trial Framework

Testing this hypothesis should proceed through a staged translational programme rather than immediately through a definitive efficacy trial of the full combination. A phase 0/1 stage should first assess formulation selection, batch-specific chemical characterisation, tolerability, pill burden, adherence, pharmacokinetics, pharmacodynamic target engagement and potential interactions. Exposure biomarkers should include, where feasible, plasma CoQ10, selenium status, urinary or plasma sulforaphane metabolites, omega-3 index, EPA oxidation/purity measures, and formulation-specific markers for polyphenol or boswellic-acid exposure. Only after formulation feasibility and safety are established should efficacy-oriented testing proceed in adults meeting the WHO post-COVID-condition definition—symptoms usually beginning within three months of infection, persisting at least two months and not explained by an alternative diagnosis—with PEM confirmed using a validated instrument and fatigue or cognitive dysfunction above a pre-specified threshold [2,4]. Stable standard care should generally be permitted rather than requiring broad medication and supplement washout, except where safety or mechanistic interpretation requires exclusion.

**Figure 2:**
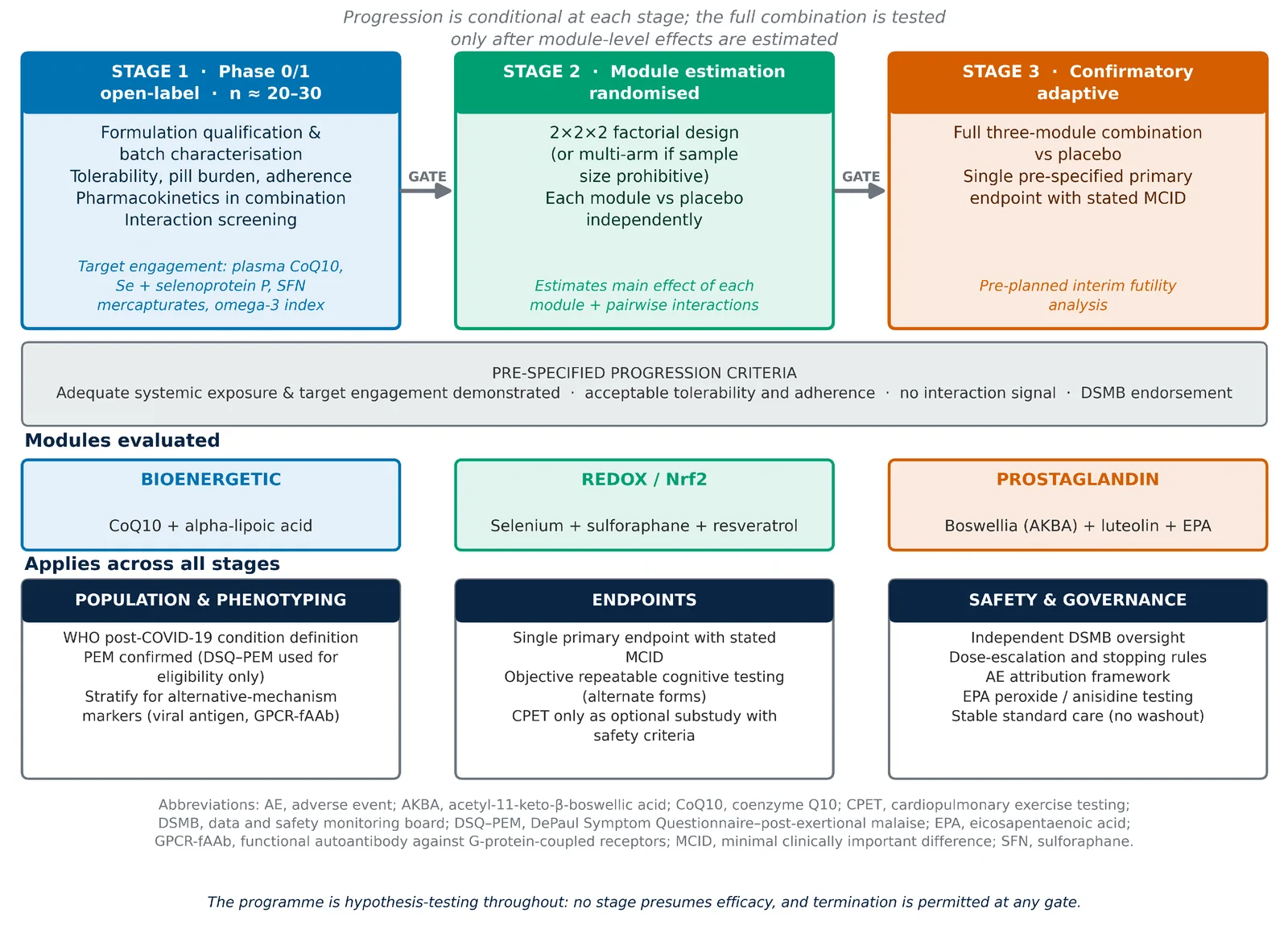
Proposed staged translational programme for evaluating the three-module nutraceutical combination. Stage 1 (phase 0/1, open-label) establishes formulation qualification, tolerability, pill burden, adherence, pharmacokinetics in combination and pharmacodynamic target engagement, using exposure biomarkers including plasma coenzyme Q10, selenium with selenoprotein P, urinary or plasma sulforaphane mercapturic acid metabolites and the erythrocyte omega-3 index. Stage 2 estimates the independent effect of each module against placebo using a 2 × 2 × 2 factorial design, or a multi-arm design where sample size is prohibitive, permitting estimation of main effects and pairwise interactions; this addresses the principal limitation of an incremental add-on ladder, which confounds module identity with position in the sequence. Stage 3 evaluates the full combination against placebo, powered on a single pre-specified primary endpoint with a stated minimal clinically important difference and incorporating pre-planned interim futility analysis. Progression between stages is conditional on pre-specified criteria (grey band). The programme is hypothesis-testing throughout: no stage presumes efficacy, and termination is permitted at any gate. Abbreviations as listed in the figure.

A single primary endpoint should be selected and justified. The DePaul Symptom Questionnaire–PEM may be useful for eligibility and phenotyping, but its responsiveness and minimal clinically important difference should be justified before use as a primary treatment endpoint. Fatigue/PEM outcomes could be supported by symptom diaries, actigraphy, wearable heart rate/HRV data and patient-reported functioning; cognitive dysfunction should be assessed using objective, repeatable tests selected to minimise practice effects. Cardiopulmonary exercise testing or exertional challenges can precipitate prolonged PEM and should therefore be optional mechanistic substudies with explicit safety criteria, dose-limiting stopping rules and post-test monitoring rather than routine requirements for all participants. A subsequent trial could use a factorial, adaptive or multi-arm design to evaluate modules independently before testing the complete combination, rather than relying only on an incremental sequence that cannot estimate redox-only effects, prostaglandin-only effects or module interactions [5,16,26]. The protocol should include predefined dose-escalation rules, adverse-event attribution procedures, adherence thresholds, blinding-integrity testing, independent data and safety monitoring, and stratification by baseline oxidative-stress/inflammatory burden. The staged programme is summarised schematically in Figure 2, which sets out the progression from phase 0/1 feasibility and target-engagement work, through module-level estimation using a factorial or multi-arm design, to confirmatory evaluation of the full combination, together with the decision points and stopping rules governing progression between stages.

A note on rehabilitation context is warranted. PEM constrains fixed-increment graded-exercise approaches—NICE defines graded-exercise therapy as establishing a baseline and then making fixed incremental increases premised on deconditioning, and nowadays most major health care organisations recommend against it in ME/CFS [63]—and therefore necessitates pacing or symptom-titrated rehabilitation strategies; this does not imply that all carefully monitored rehabilitation is contraindicated in every Long COVID phenotype. Indeed, individually symptom-titrated exercise, delivered with cautious PEM monitoring, has improved fatigue and quality of life in selected post-COVID-condition patients [64]. A nutraceutical trial should be positioned as complementary to, not a replacement for, individualised rehabilitation and standard care.

## 9. Conclusions

Post-exertional malaise and cognitive dysfunction in Long COVID may involve interacting disturbances in mitochondrial bioenergetics, redox regulation and neurovascular inflammation, with the mPGES-1/PGE_2_ component representing a particularly speculative but testable sub-hypothesis. Reframing nutraceuticals as pathway-relevant candidate interventions rather than generic antioxidants yields a coherent translational framework, but no component has demonstrated efficacy specifically for both PEM and cognitive dysfunction, no study has evaluated the complete eight-agent combination, pharmacokinetic compatibility and target engagement are unknown, and additive efficacy cannot be inferred from mechanistic complementarity. Combining several agents may add toxicity, pill burden and interpretive complexity without adding benefit. The model is therefore suitable for staged experimental evaluation—beginning with formulation, exposure, safety and biomarker studies—before any adequately powered efficacy trial is attempted.

## Figures and Tables

**Table 2:** Qualitative evidence grading for principal claims, with explicit criteria distinguishing directness, study design, consistency and risk-of-bias limitations.

Claim	Direct Long COVID Human Evidence	Indirect Human Evidence	Preclinical/Mechanistic Evidence	Overall Certainty
Mitochondrial dysfunction contributes to PEM	Moderate: direct CPET and muscle-biopsy/metabolomic evidence, with heterogeneity.	Moderate: ME/CFS/post-viral overlap.	Moderate: biologically coherent, though not specific to all Long COVID phenotypes.	Moderate
PDH flux restriction is a central Long COVID lesion	Very limited: inferred from metabolic patterns, not directly established as central.	Moderate: ME/CFS/metabolic literature.	Moderate: plausible but indirect.	Low
Persistent Nrf2–thioredoxin suppression in established Long COVID	None: persistent Nrf2–thioredoxin suppression has not been directly demonstrated in established Long COVID.	Limited: acute COVID and in vitro viral-interference evidence only.	Moderate: pathway biology well characterised; persistence in chronic Long COVID not established.	Low
mPGES-1/PGE_2_ drives PEM or cognitive dysfunction	None: no direct evidence of mPGES-1/PGE_2_-specific abnormality in target phenotype.	Limited: inflammatory/neurovascular evidence, not prostaglandin-specific.	Moderate: mPGES-1/PGE_2_ biology well characterised in inflammation generally, not in Long COVID.	Very low
CoQ10 + ALA improves Long COVID fatigue	Limited: prospective non-randomised comparison with untreated controls.	Limited: metabolic/redox support from non-Long COVID conditions.	Limited: mechanistically coherent but non-specific; oral ALA does not restore covalently attached PDH lipoylation.	Low
EPA-dominant therapy benefits Long COVID PEM/cognition	Very limited: feasibility/tolerability trial; not EPA-dominant efficacy evidence.	Moderate: cardiovascular/inflammatory evidence in different populations.	Moderate: eicosanoid and resolution biology well characterised, though not in Long COVID.	Low
Complete three-module combination attenuates PEM/cognition	None: untested.	Very limited: polytherapy rationale from mitochondrial/nutritional medicine only.	Very limited: no target-engagement, pharmacokinetic-compatibility or interaction data for the combination.	Very low

Evidence categories were applied as follows: “none” means no direct evidence in the specified population or endpoint; “very limited” means small, indirect, uncontrolled or exploratory evidence with major directness/risk-of-bias limitations; “limited” means at least one direct human study or consistent indirect human evidence but without confirmatory randomised evidence; “moderate” means multiple convergent human studies or a well-conducted direct study with remaining limitations; and “strong” means consistent, replicated evidence with high directness. Overall certainty is reported on a single four-level scale—very low, low, moderate, high—integrating directness, study design and consistency across the three evidence columns. One category only is assigned in every cell. These ratings are the author’s qualitative appraisal for this hypothesis-generating narrative review and are not derived from a formal GRADE assessment. ALA, alpha-lipoic acid; CoQ10, coenzyme Q10; EPA, eicosapentaenoic acid; ME/CFS, myalgic encephalomyelitis/chronic fatigue syndrome; mPGES-1, microsomal prostaglandin E_2_ synthase-1; Nrf2, nuclear factor erythroid 2-related factor 2; PDH, pyruvate dehydrogenase; PEM, post-exertional malaise; PGE_2_, prostaglandin E_2_.

## Data Availability

No new data were created or analysed in this study. Data sharing is not applicable to this article.

## Abbreviations

ACE2	angiotensin-converting enzyme 2
AKBA	acetyl-11-keto-β-boswellic acid
ALA	alpha-lipoic acid
AP-1	activator protein 1
ATP	adenosine triphosphate
Bos	*Boswellia serrata*
CoQ10	coenzyme Q10
COX-2	cyclooxygenase-2
CPET	cardiopulmonary exercise testing
CSF	cerebrospinal fluid
CYP1A2	cytochrome P450 1A2
DHA	docosahexaenoic acid
EPA	eicosapentaenoic acid
ETC	electron transport chain
GCL	glutamate-cysteine ligase
GPCR	G-protein-coupled receptor
HRV	heart rate variability
Keap1	Kelch-like ECH-associated protein 1
Lut	luteolin
ME/CFS	myalgic encephalomyelitis/chronic fatigue syndrome
mPGES-1	microsomal prostaglandin E_2_ synthase-1
NF-κB	nuclear factor kappa-B
NQO1	NAD(P)H quinone oxidoreductase 1
Nrf2	nuclear factor erythroid 2-related factor 2
PASC	post-acute sequelae of SARS-CoV-2
PBMC	peripheral blood mononuclear cell
PDH	pyruvate dehydrogenase
PEM	post-exertional malaise
PGE2	prostaglandin E_2_
PGH2	prostaglandin H_2_
PK	pharmacokinetic(s)
POTS	postural orthostatic tachycardia syndrome
Prdx	peroxiredoxin
PRISMA	Preferred Reporting Items for Systematic Reviews and Meta-Analyses
RCT	randomised controlled trial
ROS	reactive oxygen species
RSV	resveratrol
SANRA	Scale for the Assessment of Narrative Review Articles
SARS-CoV-2	severe acute respiratory syndrome coronavirus 2
Se	selenium
SFN	sulforaphane
SIRT1	sirtuin 1
SPMs	specialised pro-resolving mediators
Trx	thioredoxin
TrxR	thioredoxin reductase
TrxR1	thioredoxin reductase 1
UL	tolerable upper intake level
WHO	World Health Organisation
